# Ferrocen­yl(meth­yl)diphenyl­silane

**DOI:** 10.1107/S1600536811014796

**Published:** 2011-04-29

**Authors:** Yu-Peng Liu, Zong-Qi Li, Yong-Xia Tan, Zhi-Jie Zhang

**Affiliations:** a Beijing National Laboratory for Molecular Sciences (BNLMS), Institute of Chemistry, Chinese Academy of Sciences, Beijing 100190, People’s Republic of China

## Abstract

In the title mol­ecule, [Fe(C_5_H_5_)(C_18_H_17_Si)], the distances of the Fe atom from the centroids of the unsubstituted and substituted cyclo­penta­dienyl (Cp) rings are 1.651 (1) and 1.646 (1) Å, respectively. The dihedral angle between the two Cp rings is 3.20 (17)°. The crystal packing is mainly stabilized by van der Waals forces.

## Related literature

For applications of transition metal compounds derived from ferrocene as catalysts, see: Togni & Hayashi (1994 ▶); and as biomolecules, see: Stepnicka (2008 ▶). For the preparation of ferrocenyl lithium, see: Rautz *et al.* (2001 ▶); and of analogues of the title compound, see: Herberhold *et al.* (2002 ▶).
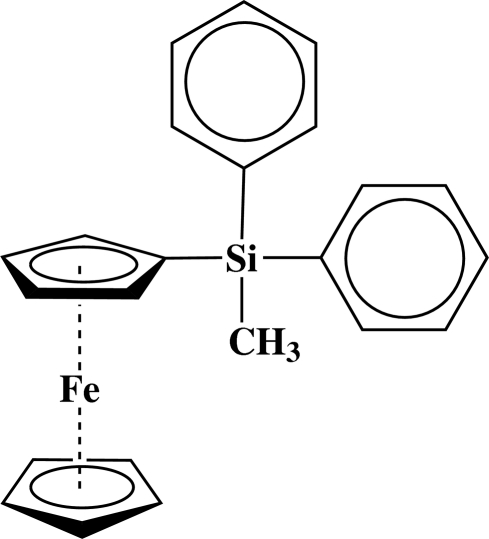


## Experimental

### 

#### Crystal data

[Fe(C_5_H_5_)(C_18_H_17_Si)]
*M* *_r_* = 382.35
Monoclinic, 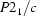

*a* = 7.4318 (15) Å
*b* = 17.795 (4) Å
*c* = 14.367 (3) Å
β = 100.408 (4)°
*V* = 1868.8 (7) Å^3^
*Z* = 4
Mo *K*α radiation
μ = 0.87 mm^−1^
*T* = 173 K
0.28 × 0.26 × 0.13 mm

#### Data collection

Rigaku MM007-HF CCD (Saturn 724+) diffractometer
Absorption correction: multi-scan (*CrystalClear*; Rigaku, 2007 ▶) *T*_min_ = 0.792, *T*_max_ = 0.895
16474 measured reflections
4265 independent reflections
3998 reflections with *I* > 2σ(*I*)
*R*_int_ = 0.047

#### Refinement

*R*[*F* ^2^ > 2σ(*F* ^2^)] = 0.049
*wR*(*F* ^2^) = 0.105
*S* = 1.18
4265 reflections
227 parameters
H-atom parameters constrained
Δρ_max_ = 0.35 e Å^−3^
Δρ_min_ = −0.25 e Å^−3^

### 

Data collection: *CrystalClear* (Rigaku, 2007 ▶); cell refinement: *CrystalClear*; data reduction: *CrystalClear*; program(s) used to solve structure: *SHELXS97* (Sheldrick, 2008 ▶); program(s) used to refine structure: *SHELXL97* (Sheldrick, 2008 ▶); molecular graphics: *XP* in *SHELXTL* (Sheldrick, 2008 ▶); software used to prepare material for publication: *SHELXL97*.

## Supplementary Material

Crystal structure: contains datablocks global, I. DOI: 10.1107/S1600536811014796/mw2007sup1.cif

Structure factors: contains datablocks I. DOI: 10.1107/S1600536811014796/mw2007Isup2.hkl

Additional supplementary materials:  crystallographic information; 3D view; checkCIF report

## Figures and Tables

**Fig. 1.**
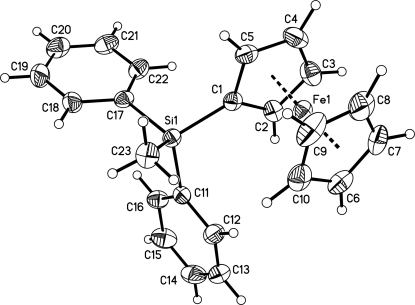
View of the title compound with 50% probability displacement ellipsoids and the atom-numbering scheme.

## supplementary crystallographic information

### Comment

Transition metal compounds derived from ferrocene have attracted considerable interest due to their applications in many fields such as catalysis (Togni & Hayashi, 1994) and biomolecules (Stepnicka, 2008). In this paper we report the synthesis and crystal structure of the title compound. In the ferrocene unit, the distances of the Fe atom from the centroids of the unsubstituted and substituted cyclopentadienyl (Cp) rings are 1.651 (1) and 1.646 (1) Å, respectively. The internal ring angle at the substituted C is smaller than the other internal ring angles. The dihedral angle between the two cyclopentadienyl rings is 3.20 (17)°. The crystal packing is mainly stabilized by van der Waals forces.

### Experimental

The preparations of FcLi and the title compound are similar to those previously reported (Rautz *et al.*, 2001; Herberhold *et al.*, 2002). Ferrocene (2.00 g, 26.88 mmol) was dissolved in 12 ml of anhydrous tetrahydrofuran (THF). In the course of 15 min a solution of 10.8 mmol *t*-BuLi (7.16 ml of a 1.5 *M n*-pentane solution) was added dropwise at 0°C. *n*-Hexane (16 ml) was then added and the solution was kept at -78°C for 15 min before the orange precipitate of FcLi was filtered off. The precipitate was washed with small portions of *n*-hexane. The FcLi was dissolved in THF (15 ml) and was added to a solution of chloromethyldiphenylsilane (2.2 g, 9.45 mmol) in *n*-hexane (20 ml) at 0°C and then stirred over night at room temperature. The precipitate was filtered off and the solvent was evaporated under vacuum. the orange residue was purified by recrystallization from *n*-hexane to give 3.26 g of yellow product in 82% yield.

### Refinement

All the H atoms were discernible in the difference electron density maps. Nevertheless, all the H atoms were constrained by the riding-hydrogen formalism with *U*_iso_(H) = 1.2*U*_eq_(C_aryl_ or _cyclopentadienyl_) or *U*_iso_(H) = 1.5*U*_eq_(C_methyl_). The C—H distances were constrained to 0.95 Å for the aryl H atoms, 0.98 Å for the the methyl H atoms and 1.00 Å for the cyclopentadienyl H atoms respectively.

### Crystal data

**Table:**

[Fe(C_5_H_5_)(C_18_H_17_Si)]	*F*(000) = 800
*M**_r_* = 382.35	*D*_x_ = 1.359 Mg m^−^^3^
Monoclinic, *P*2_1_/*c*	Mo *K*α radiation, λ = 0.71073 Å
Hall symbol: -P 2ybc	Cell parameters from 6544 reflections
*a* = 7.4318 (15) Å	θ = 1.8–27.5°
*b* = 17.795 (4) Å	µ = 0.87 mm^−^^1^
*c* = 14.367 (3) Å	*T* = 173 K
β = 100.408 (4)°	Block, yellow
*V* = 1868.8 (7) Å^3^	0.28 × 0.26 × 0.13 mm
*Z* = 4	

### Data collection

**Table:**

Rigaku MM007-HF CCD (Saturn 724+) diffractometer	4265 independent reflections
Radiation source: rotating anode	3998 reflections with *I* > 2σ(*I*)
Confocal	*R*_int_ = 0.047
ω scans at fixed χ = 45°	θ_max_ = 27.5°, θ_min_ = 1.8°
Absorption correction: multi-scan (*CrystalClear*; Rigaku, 2007)	*h* = −9→9
*T*_min_ = 0.792, *T*_max_ = 0.895	*k* = −22→23
16474 measured reflections	*l* = −18→18

### Refinement

**Table:**

Refinement on *F*^2^	Primary atom site location: structure-invariant direct methods
Least-squares matrix: full	Secondary atom site location: difference Fourier map
*R*[*F*^2^ > 2σ(*F*^2^)] = 0.049	Hydrogen site location: inferred from neighbouring sites
*wR*(*F*^2^) = 0.105	H-atom parameters constrained
*S* = 1.18	*w* = 1/[σ^2^(*F*_o_^2^) + (0.0318*P*)^2^ + 1.4337*P*] where *P* = (*F*_o_^2^ + 2*F*_c_^2^)/3
4265 reflections	(Δ/σ)_max_ < 0.001
227 parameters	Δρ_max_ = 0.35 e Å^−^^3^
0 restraints	Δρ_min_ = −0.25 e Å^−^^3^

### Special details

**Table:**

Geometry. All e.s.d.'s (except the e.s.d. in the dihedral angle between two l.s. planes) are estimated using the full covariance matrix. The cell e.s.d.'s are taken into account individually in the estimation of e.s.d.'s in distances, angles and torsion angles; correlations between e.s.d.'s in cell parameters are only used when they are defined by crystal symmetry. An approximate (isotropic) treatment of cell e.s.d.'s is used for estimating e.s.d.'s involving l.s. planes. X1A and X1B are the centroids of the substituted and unsubstituted cyclopentadienyl (Cp) rings, respectively.
Refinement. Refinement of *F*^2^ against ALL reflections. The weighted *R*-factor *wR* and goodness of fit *S* are based on *F*^2^, conventional *R*-factors *R* are based on *F*, with *F* set to zero for negative *F*^2^. The threshold expression of *F*^2^ > σ(*F*^2^) is used only for calculating *R*-factors(gt) *etc*. and is not relevant to the choice of reflections for refinement. *R*-factors based on *F*^2^ are statistically about twice as large as those based on *F*, and *R*-factors based on ALL data will be even larger.

### Fractional atomic coordinates and isotropic or equivalent isotropic displacement parameters (Å^2^)

**Table:**

	*x*	*y*	*z*	*U*_iso_*/*U*_eq_	
Fe1	0.26293 (4)	0.334745 (19)	0.53570 (2)	0.02536 (11)	
Si1	0.45588 (9)	0.15904 (4)	0.48452 (4)	0.02365 (15)	
C1	0.4252 (3)	0.26273 (13)	0.47397 (15)	0.0248 (5)	
C2	0.5333 (3)	0.31775 (14)	0.53173 (18)	0.0288 (5)	
H2A	0.6307	0.3066	0.5877	0.035*	
C3	0.4780 (4)	0.39057 (15)	0.4979 (2)	0.0367 (6)	
H3A	0.5306	0.4393	0.5249	0.044*	
C4	0.3346 (4)	0.38192 (16)	0.41877 (19)	0.0391 (6)	
H4A	0.2682	0.4236	0.3803	0.047*	
C5	0.3012 (3)	0.30396 (15)	0.40376 (16)	0.0311 (5)	
H5A	0.2063	0.2814	0.3532	0.037*	
C6	0.2323 (4)	0.33179 (17)	0.67424 (18)	0.0378 (6)	
H6A	0.3321	0.3234	0.7300	0.045*	
C7	0.1755 (4)	0.40192 (17)	0.6337 (2)	0.0443 (7)	
H7A	0.2273	0.4520	0.6558	0.053*	
C8	0.0317 (4)	0.3887 (2)	0.5559 (2)	0.0494 (8)	
H8A	−0.0361	0.4280	0.5135	0.059*	
C9	0.0006 (4)	0.3108 (2)	0.5492 (2)	0.0472 (8)	
H9A	−0.0929	0.2848	0.5011	0.057*	
C10	0.1244 (4)	0.27564 (17)	0.62260 (19)	0.0390 (6)	
H10A	0.1343	0.2204	0.6352	0.047*	
C11	0.6161 (3)	0.13831 (13)	0.59914 (16)	0.0257 (5)	
C12	0.5675 (4)	0.15244 (15)	0.68696 (17)	0.0344 (6)	
H12A	0.4517	0.1743	0.6892	0.041*	
C13	0.6844 (4)	0.13532 (16)	0.77123 (18)	0.0395 (6)	
H13A	0.6478	0.1452	0.8301	0.047*	
C14	0.8527 (4)	0.10412 (16)	0.76933 (19)	0.0413 (7)	
H14A	0.9327	0.0924	0.8269	0.050*	
C15	0.9057 (4)	0.08981 (17)	0.6838 (2)	0.0409 (6)	
H15A	1.0223	0.0684	0.6824	0.049*	
C16	0.7881 (3)	0.10685 (14)	0.59942 (17)	0.0311 (5)	
H16A	0.8258	0.0968	0.5409	0.037*	
C17	0.5722 (3)	0.12657 (14)	0.38585 (15)	0.0256 (5)	
C18	0.5443 (4)	0.05473 (14)	0.34648 (17)	0.0322 (5)	
H18A	0.4606	0.0216	0.3682	0.039*	
C19	0.6371 (4)	0.03118 (16)	0.2761 (2)	0.0424 (7)	
H19A	0.6152	−0.0176	0.2497	0.051*	
C20	0.7603 (4)	0.07783 (17)	0.24421 (18)	0.0401 (6)	
H20A	0.8248	0.0611	0.1967	0.048*	
C21	0.7898 (4)	0.14908 (17)	0.28160 (18)	0.0373 (6)	
H21A	0.8741	0.1818	0.2596	0.045*	
C22	0.6961 (3)	0.17263 (15)	0.35131 (17)	0.0322 (5)	
H22A	0.7172	0.2219	0.3764	0.039*	
C23	0.2338 (3)	0.10882 (16)	0.47803 (19)	0.0353 (6)	
H23A	0.1518	0.1222	0.4190	0.053*	
H23B	0.1777	0.1234	0.5321	0.053*	
H23C	0.2553	0.0545	0.4796	0.053*	

### Atomic displacement parameters (Å^2^)

**Table:**

	*U*^11^	*U*^22^	*U*^33^	*U*^12^	*U*^13^	*U*^23^
Fe1	0.02179 (18)	0.0283 (2)	0.02612 (18)	0.00286 (14)	0.00470 (13)	−0.00229 (13)
Si1	0.0220 (3)	0.0261 (3)	0.0235 (3)	0.0012 (3)	0.0060 (2)	−0.0008 (2)
C1	0.0218 (11)	0.0294 (12)	0.0250 (11)	0.0034 (10)	0.0092 (9)	0.0005 (9)
C2	0.0211 (11)	0.0294 (12)	0.0372 (13)	−0.0009 (10)	0.0089 (9)	−0.0002 (10)
C3	0.0326 (14)	0.0277 (13)	0.0524 (16)	−0.0049 (11)	0.0147 (12)	0.0019 (11)
C4	0.0411 (15)	0.0362 (15)	0.0427 (15)	0.0083 (13)	0.0149 (12)	0.0140 (11)
C5	0.0309 (13)	0.0387 (14)	0.0241 (11)	0.0042 (12)	0.0062 (9)	0.0028 (10)
C6	0.0326 (14)	0.0552 (18)	0.0264 (12)	0.0024 (13)	0.0074 (10)	−0.0059 (11)
C7	0.0482 (17)	0.0425 (16)	0.0460 (16)	0.0049 (14)	0.0187 (13)	−0.0158 (13)
C8	0.0394 (16)	0.066 (2)	0.0445 (16)	0.0295 (16)	0.0113 (13)	−0.0023 (14)
C9	0.0194 (12)	0.080 (2)	0.0428 (16)	−0.0023 (14)	0.0086 (11)	−0.0226 (15)
C10	0.0366 (14)	0.0426 (16)	0.0438 (15)	−0.0039 (13)	0.0231 (12)	−0.0059 (12)
C11	0.0281 (12)	0.0230 (11)	0.0263 (11)	0.0002 (10)	0.0061 (9)	0.0009 (9)
C12	0.0384 (14)	0.0374 (14)	0.0279 (12)	0.0070 (12)	0.0069 (10)	0.0006 (10)
C13	0.0522 (17)	0.0406 (15)	0.0254 (12)	0.0000 (14)	0.0062 (11)	0.0005 (11)
C14	0.0408 (15)	0.0460 (16)	0.0329 (14)	−0.0042 (13)	−0.0051 (11)	0.0114 (11)
C15	0.0285 (13)	0.0491 (17)	0.0438 (15)	0.0051 (13)	0.0028 (11)	0.0130 (13)
C16	0.0285 (12)	0.0355 (13)	0.0300 (12)	0.0003 (11)	0.0072 (10)	0.0052 (10)
C17	0.0241 (11)	0.0309 (12)	0.0214 (10)	0.0047 (10)	0.0029 (8)	0.0019 (9)
C18	0.0378 (14)	0.0273 (12)	0.0338 (13)	0.0018 (11)	0.0124 (11)	0.0004 (10)
C19	0.0571 (18)	0.0329 (14)	0.0411 (15)	0.0067 (14)	0.0194 (13)	−0.0053 (11)
C20	0.0407 (15)	0.0511 (17)	0.0324 (13)	0.0126 (14)	0.0168 (11)	0.0004 (12)
C21	0.0312 (13)	0.0524 (17)	0.0299 (13)	−0.0037 (13)	0.0101 (10)	0.0023 (11)
C22	0.0304 (13)	0.0375 (14)	0.0280 (12)	−0.0045 (11)	0.0036 (10)	−0.0042 (10)
C23	0.0275 (13)	0.0377 (15)	0.0412 (14)	−0.0035 (11)	0.0076 (11)	−0.0043 (11)

### Geometric parameters (Å, °)

**Table:**

Fe1—C4	2.033 (3)	C8—H8A	1.0000
Fe1—C8	2.035 (3)	C9—C10	1.414 (4)
Fe1—C3	2.037 (3)	C9—H9A	1.0000
Fe1—C9	2.039 (3)	C10—H10A	1.0000
Fe1—C7	2.040 (3)	C11—C16	1.395 (3)
Fe1—C5	2.042 (2)	C11—C12	1.397 (3)
Fe1—C2	2.043 (2)	C12—C13	1.390 (4)
Fe1—C6	2.045 (3)	C12—H12A	0.9500
Fe1—C10	2.047 (3)	C13—C14	1.374 (4)
Fe1—C1	2.066 (2)	C13—H13A	0.9500
Si1—C1	1.862 (2)	C14—C15	1.381 (4)
Si1—C23	1.864 (3)	C14—H14A	0.9500
Si1—C17	1.880 (2)	C15—C16	1.394 (3)
Si1—C11	1.886 (2)	C15—H15A	0.9500
C1—C2	1.432 (3)	C16—H16A	0.9500
C1—C5	1.438 (3)	C17—C22	1.390 (3)
C2—C3	1.419 (3)	C17—C18	1.398 (3)
C2—H2A	1.0000	C18—C19	1.388 (3)
C3—C4	1.419 (4)	C18—H18A	0.9500
C3—H3A	1.0000	C19—C20	1.374 (4)
C4—C5	1.419 (4)	C19—H19A	0.9500
C4—H4A	1.0000	C20—C21	1.379 (4)
C5—H5A	1.0000	C20—H20A	0.9500
C6—C10	1.406 (4)	C21—C22	1.384 (4)
C6—C7	1.409 (4)	C21—H21A	0.9500
C6—H6A	1.0000	C22—H22A	0.9500
C7—C8	1.420 (4)	C23—H23A	0.9800
C7—H7A	1.0000	C23—H23B	0.9800
C8—C9	1.406 (5)	C23—H23C	0.9800
			
C1—Si1—C23	112.13 (11)	C13—C12—C11	121.6 (3)
C1—Si1—C17	108.03 (10)	C13—C12—H12A	119.2
C23—Si1—C17	109.78 (11)	C11—C12—H12A	119.2
C1—Si1—C11	108.27 (10)	C14—C13—C12	119.9 (3)
C23—Si1—C11	111.31 (11)	C14—C13—H13A	120.0
C17—Si1—C11	107.13 (10)	C12—C13—H13A	120.0
C2—C1—C5	106.2 (2)	C13—C14—C15	120.0 (2)
C2—C1—Si1	125.65 (18)	C13—C14—H14A	120.0
C5—C1—Si1	128.02 (18)	C15—C14—H14A	120.0
C3—C2—C1	109.2 (2)	C14—C15—C16	119.9 (3)
C3—C2—H2A	125.4	C14—C15—H15A	120.0
C1—C2—H2A	125.4	C16—C15—H15A	120.0
C2—C3—C4	107.7 (2)	C15—C16—C11	121.4 (2)
C2—C3—H3A	126.1	C15—C16—H16A	119.3
C4—C3—H3A	126.1	C11—C16—H16A	119.3
C3—C4—C5	108.2 (2)	C22—C17—C18	117.0 (2)
C3—C4—H4A	125.9	C22—C17—Si1	120.84 (19)
C5—C4—H4A	125.9	C18—C17—Si1	122.10 (18)
C4—C5—C1	108.7 (2)	C19—C18—C17	120.9 (2)
C4—C5—H5A	125.7	C19—C18—H18A	119.5
C1—C5—H5A	125.7	C17—C18—H18A	119.5
C10—C6—C7	108.2 (3)	C20—C19—C18	120.6 (3)
C10—C6—H6A	125.9	C20—C19—H19A	119.7
C7—C6—H6A	125.9	C18—C19—H19A	119.7
C6—C7—C8	107.8 (3)	C19—C20—C21	119.6 (2)
C6—C7—H7A	126.1	C19—C20—H20A	120.2
C8—C7—H7A	126.1	C21—C20—H20A	120.2
C9—C8—C7	107.9 (3)	C20—C21—C22	119.6 (3)
C9—C8—H8A	126.1	C20—C21—H21A	120.2
C7—C8—H8A	126.1	C22—C21—H21A	120.2
C8—C9—C10	108.0 (3)	C21—C22—C17	122.2 (2)
C8—C9—H9A	126.0	C21—C22—H22A	118.9
C10—C9—H9A	126.0	C17—C22—H22A	118.9
C6—C10—C9	108.1 (3)	Si1—C23—H23A	109.5
C6—C10—H10A	125.9	Si1—C23—H23B	109.5
C9—C10—H10A	125.9	H23A—C23—H23B	109.5
C16—C11—C12	117.1 (2)	Si1—C23—H23C	109.5
C16—C11—Si1	120.98 (17)	H23A—C23—H23C	109.5
C12—C11—Si1	121.88 (19)	H23B—C23—H23C	109.5
			
C23—Si1—C1—C2	135.4 (2)	C17—Si1—C11—C12	−177.0 (2)
C17—Si1—C1—C2	−103.5 (2)	C16—C11—C12—C13	−0.6 (4)
C11—Si1—C1—C2	12.2 (2)	Si1—C11—C12—C13	178.4 (2)
C23—Si1—C1—C5	−49.7 (2)	C11—C12—C13—C14	0.4 (4)
C17—Si1—C1—C5	71.4 (2)	C12—C13—C14—C15	0.0 (4)
C11—Si1—C1—C5	−172.9 (2)	C13—C14—C15—C16	−0.2 (4)
C5—C1—C2—C3	−0.2 (3)	C14—C15—C16—C11	−0.1 (4)
Si1—C1—C2—C3	175.60 (17)	C12—C11—C16—C15	0.4 (4)
C1—C2—C3—C4	0.2 (3)	Si1—C11—C16—C15	−178.6 (2)
C2—C3—C4—C5	0.0 (3)	C1—Si1—C17—C22	31.9 (2)
C3—C4—C5—C1	−0.1 (3)	C23—Si1—C17—C22	154.5 (2)
C2—C1—C5—C4	0.2 (3)	C11—Si1—C17—C22	−84.5 (2)
Si1—C1—C5—C4	−175.47 (17)	C1—Si1—C17—C18	−150.6 (2)
C10—C6—C7—C8	−0.3 (3)	C23—Si1—C17—C18	−28.0 (2)
C6—C7—C8—C9	0.1 (3)	C11—Si1—C17—C18	93.0 (2)
C7—C8—C9—C10	0.1 (3)	C22—C17—C18—C19	0.0 (4)
C7—C6—C10—C9	0.4 (3)	Si1—C17—C18—C19	−177.6 (2)
C8—C9—C10—C6	−0.3 (3)	C17—C18—C19—C20	0.7 (4)
C1—Si1—C11—C16	−114.3 (2)	C18—C19—C20—C21	−1.0 (4)
C23—Si1—C11—C16	122.0 (2)	C19—C20—C21—C22	0.5 (4)
C17—Si1—C11—C16	2.0 (2)	C20—C21—C22—C17	0.2 (4)
C1—Si1—C11—C12	66.7 (2)	C18—C17—C22—C21	−0.5 (4)
C23—Si1—C11—C12	−56.9 (2)	Si1—C17—C22—C21	177.1 (2)
